# Investigating the Epidemiology of *Haemonchus contortus*, Using Traditional Faecal Diagnostics Supported by Deep‐Amplicon Sequencing, in an Outbreak of Haemonchosis in a Scottish Pedigree Sheep Flock

**DOI:** 10.1155/crve/5558878

**Published:** 2026-08-06

**Authors:** A. Luque Castro, N. D. Sargison, A. Morrison, D. J. Bartley, M. Abbas, A. I. Macrae, F. R. Murdoch, R. F. Kelly

**Affiliations:** ^1^ Royal (Dick) School of Veterinary Studies and Roslin Institute, University of Edinburgh, Midlothian, UK, ed.ac.uk; ^2^ Ross University School of Veterinary Medicine, Basseterre, Saint Kitts and Nevis, rossu.edu; ^3^ Moredun Research Institute, Pentlands Science Park, Penicuik, UK, moredun.org.uk; ^4^ School of Veterinary Medicine, University of Surrey, Surrey, UK, surrey.ac.uk

## Abstract

This case report describes an investigation of an outbreak of haemonchosis in a Scottish pedigree sheep flock. The case occurred in a small lowland flock of pedigree Spotted Dutch sheep (*n* = 16) grazing pasture previously grazed by alpacas and horses. In 2024, despite routine anthelmintic treatments, clinical signs in the sheep led to suspicions and subsequent confirmation of acute haemonchosis. As part of clinical investigations, traditional faecal parasitology was combined with species identification using peanut agglutinin staining of GIN eggs and ITS‐2 deep‐amplicon genomic sequencing of first‐stage larvae. Using these techniques, *Haemonchus contortus* was identified as the predominant GIN, and FAMACHA scoring was used to assess the level of anaemia for targeted selective treatment of affected animals. Additionally, ITS‐2 deep‐amplicon genomic sequencing described the GIN species profile of different animal management groups to further understand *H. contortus* epidemiology and inform control practices against future outbreaks. This case highlights the benefits of combining traditional faecal egg count diagnostics with GIN species identification techniques to better understand *H. contortus* epidemiology, thereby promoting sustainable control practices against haemonchosis.

## 1. Introduction

*Haemonchus contortus* is a parasitic gastrointestinal nematode (GIN) of sheep and goats (small ruminants) and can be a coinfection with other common GIN, contributing to classical parasitic gastroenteritis (PGE) [1]. Predominance of *H. contortus* over other GIN can occur, as females are prolific egg layers producing ~5000–15,000 eggs per day, compared with only hundreds per day produced by females of other common GIN species [2]. Unlike other GIN in small ruminants, *H. contortus* blood feeds within the digestive tract of hosts and is also known as the ‘barber′s pole worm’ due to the blood‐filled appearance of adult worms. Subsequently, *H. contortus* can result in a specific disease presentations termed ‘haemonchosis’ with clinical signs (e.g. anaemia) associated with its blood‐feeding behaviour rather than those associated with classical PGE in mixed GIN infections (e.g. diarrhoea and associated weight loss). Although *H. contortus* is a GIN of small ruminants, it can infect other grazing species. In UK settings, although cattle or horses can be infected, they are unlikely to be involved in transmission (e.g. they are likely spillover hosts) or develop disease. Other domestic grazing species (e.g. camelids) can be a reservoir and susceptible to disease. In some settings, grazing wildlife species (e.g. deer) may potentially act as a reservoir of infection to small ruminants [3]. Therefore, cograzing host species should be considered when investigating outbreaks and looking for sources of infection [4].

Globally, the prevalence of haemonchosis has been highest in tropical and subtropical areas, where warm (optimal 20°C–30°C) and humid conditions favour rapid development and the survival of free‐living stages in the environment [5]. However, *H. contortus* has been shown to be adaptable to a variety of environments, giving rise to unpredictable epidemiology and unexpected disease outbreaks in cooler settings [2, 6–8]. For example, *H. contortus* is highly polymorphic, conferring a high level of genetic adaptation to drug exposure [9] and the ability to overwinter on pasture at a range of temperatures [6]. These mechanisms are the key for *H. contortus* survival in colder temperate regions such as the United Kingdom and the rest of Northern Europe, leading to disease outbreaks out of season and in unexpected geographical locations [10, 11]. Understanding the dynamics of particular GIN species within individual farms could aid prevention of future outbreaks of haemonchosis. Using diagnostic tools that identify GIN species present in suspected haemonchosis outbreaks could aid the understanding of *H. contortus* epidemiology to inform immediate control practices and aid future prevention of disease outbreaks.

This case report highlights the potential benefits of using ITS‐2 deep‐amplicon genomic sequencing to identify GIN species present in an investigation of an outbreak of haemonchosis in a Scottish smallholder pedigree sheep flock.

## 2. Case Report

### 2.1. Flock Context

The case presented in a lowland flock of pedigree Spotted Dutch sheep situated in Southeast Scotland. The flock produces breeding animals and is used to teach local school students about animal husbandry. The last new animal was introduced 3 years previously (a single ram, unknown quarantine history) and was otherwise a closed group of seven breeding ewes, four hoggs (yearling females) and five rams. Each year, ewes are mated in early autumn to be lambed in February–March. Pregnant ewes are housed in November and kept inside for 2–3 months after lambing. Ewes and lambs are turned out between April and June, depending on weather conditions and lambs weaning in June–July.

Approximately 1 ha of grazing land was available each year for the flock, which was split into five permanent paddocks that had only been grazed by horses for the previous 1 years. During the grazing season of summer in 2023, ewes, hoggs and rams were managed in these paddocks as separate groups. Contrasting to previous years, two alpacas also utilised this grazing. In 2024, ewes were kept inside until early May. Six goats were also on the farm but did not share this or any other grazing with the sheep, alpacas or horses. Due to the numerous animal species groups and limited acreage of the entire farm (~20 acres), it was not considered to be feasible to rest pastures and provide clean grazing fields for GIN control. To manage larval challenge from pasture, a novel approach of removing sheep faeces from pasture was conducted on a weekly basis from all five paddocks by local school students.

Breeding ewes were routinely weighed and treated with a levamisole drench (7.5 mg/kg body weight [BW]) after lambing and with an albendazole drench (5.0 mg/kg BW) before housing. In years when national surveillance forecasting indicated a risk of nematodirosis, lambs were treated with albendazole drench (5.0 mg/kg BW) in May or June. Between 2021 and 2024, routine faecal worm egg count (FEC) monitoring had been low for all groups (modified McMaster method: < 50 eggs per gram [epg]). Prior to 2024, haemonchosis had never been diagnosed in the flock, and *Fasciola hepatica* eggs had never been detected via faecal sedimentation.

### 2.2. Clinical Presentation

In May 2024, the keeper identified that the ewes were in poor body condition compared with prelambing assessment 4–5 months previously. All ewes were individually body condition scored (BCS). BCS among the ewes (anecdotally ≤ 2) were below expected targets for this stage of production, whereas rams and hoggs maintained moderate scores (2.5). All ewes were subsequently treated with an albendazole drench (5.0 mg/kg BW) to cover for chronic fasciolosis or PGE.

### 2.3. Clinical and Laboratory Diagnostics

Within 24 h of treatment with albendazole, one thin ewe (Ewe 1: BCS 1.5) exhibited severe clinical signs including pale mucous membranes, evidenced by a high FAMACHA score (FAMACHA score of 1–5, with 1 implying *no anaemia* and 5 implying *severe anaemia*), submandibular oedema, and a high heart rate (120 bpm). No other abnormalities were found by clinical examination. The same day, blood samples were taken by jugular venepuncture for haematology and biochemistry (EDTA and lithium heparin, respectively). Haematology results revealed a mild neutrophilia, lymphopenia, reduced red cell count, very low haematocrit, low haemoglobin concentration and reticulocytosis (Table 1). These results reflect acute inflammation and regenerative anaemia, with the latter caused by acute haemorrhage or haemolysis. Additionally, biochemistry results showed hypoproteinaemia along with a mildly elevated gamma‐glutamyl transferase (GGT) and glutamate dehydrogenase (GLDH) liver enzymes (Table 1). Although these biochemical changes are less specific, they supported a diagnosis of acute to chronic parasitism.

**Table 1:** Sick ewe haematology and biochemistry results.

Parameter	Result	Reference range
Haematology		
White blood cells (total)	7.40 x10e^9^/L	4.0–10.0 x 10e^9^/L
Neutrophils (segmented)	∗75.0%	10.0%–50.0%
	∗5.5 x10e^9^/L	0.4–5.0 x 10e^9^/L
Neutrophils (nonsegmented)	0.0%	0.0%–1.0%
	0.0 x10e^9^/L	0.0–0.1 x 10e^9^/L
Lymphocytes	∗22.0%	50.0%–75.0%
	1.6 x10e^9^/L	1.6–7.5 x 10e^9^/L
Monocytes	3.0%	0.0%–6.0%
	0.2 x10e^9^/L	0.0–0.6 x 10e^9^/L
Eosinophils	0.0%	0.0%–10.0%
	0.0 x10e^9^/L	0.0–1.0 x 10e^9^/L
Basophils	0.0%	0.0–3.0%
	0.0 x10e^9^/L	0.0–0.3 x 10e^9^/L
Red blood cells (total)	∗2.24 x10e^12^/L	5.0–11.0 x 10e^12^/L
Haematocrit	∗0.1 L/L	0.3–0.4 L/L
Haemoglobin	∗2.2 g/dL	8.0–14.0 g/dL
Mean corpuscular volume	35.1 fL	28.0–48.0 fL
Mean corpuscular haemoglobin concentration	∗25.3 g/dL	31.0–38.0 g/dL
Platelets (automated count)	742.0 x10e^9^/L	250.0–750.0 x10e^9^/L
Biochemistry		
Total protein	∗39.8 g/L	73.0–89.0 g/L
Albumin	∗14.3 g/L	24.0–35.0 g/L
Globulin	∗25.5 g/L	34.0–55.0 g/L
GGT	∗227 u/L	34.0–100.0 u/L
GLDH	∗19 u/L	1.0–12.0 u/L

*Note:* Asterisk “∗” denotes abnormal values.

A FEC on 2 g of faeces was conducted using the saturated saline (NaCl) centrifugal flotation cuvette technique, with a detection threshold of 3 epg [12]. In the sick ewe, with a *Trichostrongylus* species egg count of > 10,000 epg, acute haemonchosis was suspected. To estimate the proportion of *H. contortus* eggs present, the faecal sample was processed within 4 days with differential fluorescein isothiocyanate–labelled peanut agglutinin (PNA) staining of *Trichostrongylus* eggs and examined under fluorescent microscopy [13]. As 98% of eggs fluoresced, indicating *H. contortus*, this was consistent with a primary diagnosis of acute haemonchosis in the sick ewe.

At the same time, other ewes were examined, highlighting variable BCS (1.5–3), FAMACHA scores suggestive of anaemia (≥ 3) and individual FWEC of 482–7722 epg (Table 2). A pooled faecal sedimentation from 2 g of faeces from each ewe (including Ewe 1) was negative for *F. hepatica* eggs, making a differential diagnosis of chronic fasciolosis unlikely. Additionally, acute fasciolosis was deemed unlikely as mass infection of juvenile fluke in the United Kingdom usually presents August–November and had previously never been diagnosed on this farm. Therefore, a diagnosis of acute haemonchosis was deemed most likely, due to consistent signs of anaemia (FAMACHA and haematology) and confirmation of significant *H. contortus* infection (FEC with subsequent PNA staining), across the whole ewe group.

**Table 2:** Individual animal body condition scores (BCS), FAMACHA score and faecal worm egg count (FEC) scores pretreatment and posttreatment with albendazole and closantel.

Animal or group identifier	Pretreatment			Posttreatment: 14 days		Posttreatment: ~7 weeks		
	BCS	FAMACHA	FEC	FEC	FECRT	BCS	FAMACHA	FEC
Individual ewes								
Ewe 1 (sick ewe)	1.5	5	≥ 10,000 epg	108 epg	≥ 98.9%^	2.5	2	63 epg
Ewe 2	1.5	4	2412 epg	9 epg	99.6%	2	3	9 epg
Ewe 3	2	4	7722 epg	126 epg	98.4%	2	3	0 epg
Ewe 4	1.5	3	3681 epg	63 epg	98.3%	2	3	0 epg
Ewe 5	2.5	4	6300 epg	30 epg	99.5%	2.5	3	—
Ewe 6	3	3	482 epg	39 epg	91.9%	3	3	33 epg
Ewe 7	2	2	1161 epg	45 epg	96.1%	2	3	0 epg
Other animal groups								
Ewe lambs (pooled)	—	—	—	< 3 epg		—	—	—
Ram lambs (pooled)	—	—	—	< 3 epg		—	—	—
Hoggs (pooled)	—	—	—	< 50 epg∗		—	—	—
Rams (pooled)	—	—	—	< 3 epg		—	—	—
Alpaca 1	—	—	—	180 epg		—	—	—
Alpaca 2	—	—	—	267 epg				

*Note:* Em dash “—” indicates no sample available. Cuvette method sensitive to 3 epg was performed for all samples, except those with “∗” for modified McMaster method sensitive to 50 epg. “^” denotes estimated percentage faecal egg count reduction due to estimated denominator.

All other sheep groups and the alpacas did not have clinical signs for acute haemonchosis but had faecal samples collected as part of the investigation into transmission dynamics. Animal groups were sampled individually (alpacas) or pooled by group (lambs, hoggs and rams) for FECs. Pooling was conducted by combining an equal portion of faeces (2 g) from each animal and homogenised in saturated saline solution, then FEC conducted via the cuvette or modified McMaster method (Table 2). Other sheep groups (lambs, hoggs and rams) were sampled as pools for FEC assessment, all having low counts (< 50 epg). The two alpacas sampled individually notably had FECs of 180 and 267 epg.

### 2.4. Initial Treatment

As ewes were initially treated with albendazole, a narrow‐spectrum product against *H. contortus*, closantel (10 mg/kg BW) was also administered based on individual FAMACHA scoring of 4 and above (total of four animals). A FEC posttreatment for faecal egg count reduction test (FECRT) was conducted 14 days later (Table 2). The subsequent FECRT showed a mean > 95% reduction in FEC (mean: 97.5%, 95%; confidence interval: 94.9%–100%), indicating an effective anthelmintic response and improvement in clinical presentation.

### 2.5. Deep‐Amplicon Genomic Sequencing of the ITS‐2 Region (Nemabiome) Method and Results

Deep‐amplicon genomic sequencing of the ITS‐2 region (nemabiome) was employed to understand the GIN species composition and transmission dynamics among ewes, other sheep groups and alpacas. As FEC and sequencing data pooled by the animal group, it does not allow attribution of species composition to individual animals but it provides an approximation of the proportions of the GIN species present and their potential significance within each animal group. For the methodology, faecal samples used for FECs were pooled by the host group, trichostrongyle eggs were isolated, incubated to generate first‐stage larvae (L1), L1 were baermannised and washed in tap water to be stored in 70% ethanol. DNA from larvae was then extracted using a lysis solution of 1000 *μ*L of DirectPCR Lysis Reagent (Viagen, United States), 50 *μ*L of proteinase K solution (Qiagen, Germany) and 50 *μ*L of 1M dithiothreitol (DTT). Once washed in distilled water, L1 were incubated with 40 *μ*L of lysis solution at 60°C for 2 h to lyse L1s and then 85°C for 15 min to inactivate the proteinase K. Genomic DNA was stored at −20°C, until further genomic analysis as per previous publications [11, 14]. In brief, PCR amplification of the ITS‐2 region involved a universal primer developed to target conserved 5.8S and 28S coding sequences of the rDNA cistron of Clade V GIN and amplify a 311–331 bp ITS‐2 fragment in which there is a discriminatory level of GIN species‐specific variation, followed by specific PCR conditions as outlined in previous publications [14]. Amplified products were then purified, barcoded and pooled for sequencing on an Illumina MiSeq platform. The sequencing utilised a 500‐cycle kit and included quality control measures like the addition of PhiX control [15].

Postsequencing data were processed via mothur software to create high‐quality aligned sequences. These were checked against a reference GIN database (https://www.nemabiome.ca/mothur_workflow.html) for species identification and used to generate amplicon sequence variants (ASVs) [11]. Analytical work continued with sequence data manipulation in R Version 4.3.3 (https://www.R-project.org/ using the Biostrings package from Bioconductor) and phylogenetic assessments using MEGA11 [16–18]. Each resequencing step followed Illumina′s standard protocol (Supporting Material S1).

**Figure 1:**
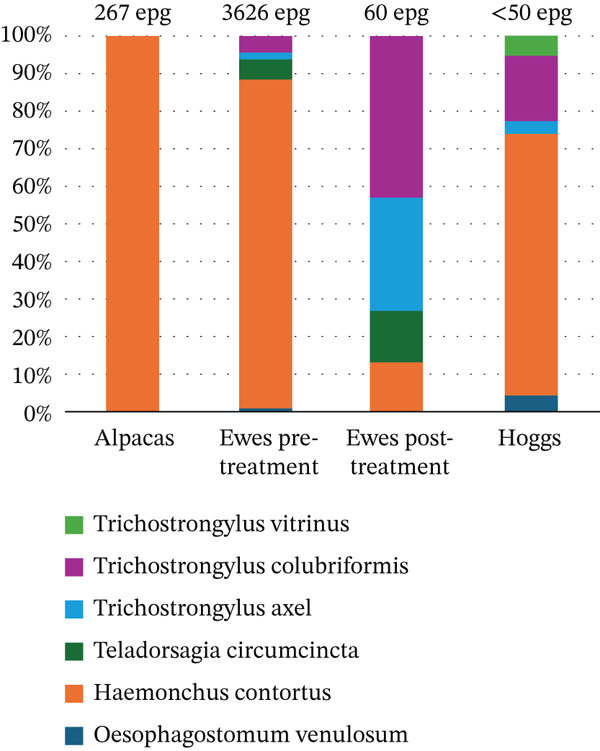
Application of the deep amplicon sequencing assay on DNA extracted from faecal egg counts of each nematode species (FEC x L3 species proportion) in the two alpacas on 08/05/2024, ewe group on 07/05/2024 pretreatment with 5 mg/kg albendazole, ewe group on 21/05/2024 posttreatment with 5 mg/kg albendazole and hogg group 08/05/2024. Note: Cuvette method sensitive to 3 epg was used as a repeated FEC for alpaca and ewe pools (after initial pool and individual FWECs, respectively). Modified McMaster method sensitive to 50 epg was used for the hogg pool. Each bar indicates a pooled animal group. Per bar, the coloured proportions represent the proportion of an individual GIN species relative to other species.

The results of deep‐amplicon genomic sequencing of the ITS‐2 region are summarised in Figure 1. Identified sequence variants for different nematode species were phylogenetically analysed, highlighting the relatedness between infecting GIN species (analysis in detail in Supporting Materials S1 and S2). Results demonstrated significant differences in nematode species across the study groups (Figure 1). In ewes, pretreatment samples showed a high predominance of *H. contortus* (88%), with smaller percentages of other common GIN species such as *Teladorsagia circumcincta* and *Trichostrongylus colubriformis*. Posttreatment, there was a major reduction in *H. contortus* (12.8%) and increases in other GIN species. In alpacas, *H. contortus* was the only GIN species detected, suggesting possible transmission links with sheep due to close relatedness. The predominance of *H. contortus* among the groups could have been potentially influenced by pasture management practices and delay in ewe exposure postlambing.

### 2.6. Clinical Outcomes

Seven weeks after treatment, ewe FAMACHA scores improved within normal limits (1–2), and all ewes attained a BCS of 2–3 (Table 2). Diagnostic monitoring using clinical assessments, FAMACHA and FWEC continues, along with planning grazing strategies considering the outcomes of case investigations. Subsequently, no health issues were reported in the flock or among the cograzing species for the remainder of 2024 and the 2025 grazing season.

## 3. Discussion

This case of ovine haemonchosis highlights the need to improve granularity of faecal diagnostics to identify GIN species present. Currently, PNA staining post FWEC, or automated FEC systems powered by generative artificial intelligence [19], can give a simple estimate of proportion of FEC associated with *H. contortus* proving useful in initial treatment decisions in this case. However, specifically combining FEC with deep‐amplicon sequencing of ITS‐2 and mitochondrial ND‐4 markers (e.g. ‘nemabiome’) has the added benefit of monitoring fluctuations in all GIN species to investigate outbreaks of disease to inform longer term control practices [20]. For example, the nemabiome has also been used to investigate GIN species in mixed species grazing systems, highlighting potential drivers for transmission from one host species to another [3]. When combined with FEC, the nemabiome has been used to longitudinally monitor GIN species and benzimidazole resistance genes within a population of ewes and lambs [21]. Such approaches could inform future clinical investigations and targeted use of anthelmintic groups to sustain treatment efficacy in the longer term. This case highlights an example of its use in a clinical investigation to inform ongoing and future preventative strategies against GIN.

In the United Kingdom, haemonchosis has been historically considered to be associated with lowlands flocks in Southern England [22]. However, in the past decade, disease has been reported across the United Kingdom with increasing incidence in the north of England and into Scotland [6, 8]. For example, Scotland′s Rural College Veterinary Services made 46 diagnoses of haemonchosis in Scottish holdings from 2012 to 2022, primarily during late summer and early autumn [23]. Of these cases, 46% were made in the last 3 years [24], associated with milder winters and warmer summers. In this case, clinical disease was detected in spring after a long period of housing, and no grazing. *H. contortus* has been present for a longer period across the United Kingdom but at much smaller proportions of total infecting GIN species [25]. As previously reported in Scotland [10], it is possible that this outbreak is a result of a previous infection carried over winter with hypobiotic L4 and subsequent maturation into adults when temperature rose in spring [26]. It is unlikely that these ewes had never encountered *H. contortus*, as they had been grazing the same paddocks for years, with only a single ram being introduced 3 years previously. However, their immunity may have waned between grazing seasons, potentially exacerbated by lack of exposure since the previous grazing season with extended housing time postlambing (2–3 months) [27].

A second hypothesis is related to grazing species sharing the same pasture. Previous grazing by alpacas and subsequent pasture management could have predisposed this outbreak. Zahid et al. [4] found that *H. contortus* was the most prevalent species in alpacas from different herds in Scotland and North England [4]. In this case, the alpacas′ FEC was 180–267 epg, and *H. contortus* predominated. These animals cograze with the sheep, and finding predominately *H. contortus* in these animals raises the question that alpacas could have been a contributing source of continued pasture contamination that went on to infect the ewes. A possible additional contributing factor is that the in‐refugia GIN population in this paddock may have been low due to the frequent faecal removal from pasture, allowing for establishment of a predominately *H. contortus* population both at pasture (in‐refugia) and in the host. Intuitively, this strategy has impact by minimising previous pasture contamination, interrupting the life cycle of GIN and reducing the ingestion of infective larvae. However, new environmental conditions may have allowed *H. contortus* to thrive and become the predominant species on the pasture. During the summer grazing season of 2023, ewes were moved on to clean pasture that had only been grazed by horses in the previous 7 years. Horses can be hosts for *H. contortus* [14], although they are likely to be spillover hosts from being grazed with preferred host species. In field conditions, GIN infections are generally mixed; therefore, different species interact and compete with each other [28, 29]. However, considering the high biotic potential of *H. contortus* (high daily egg output, ~4000 eggs/female/day and short prepatent period) may have led to a predominance of *H. contortus* in‐refugia from either previous grazing from the flock or from the alpacas. Further case‐controlled field studies are vital to test such hypotheses about dynamics with cograzing species and the influence of faecal removal, especially as the latter is a common practice in husbandry of camelids at pasture in the United Kingdom.

The presence of *H. contortus* and development of haemonchosis has implications for the future GIN control in this flock and subsequent pasture management. This flock has the advantages of using targeted approaches due to its small size, high ratio of farm personnel and elsewhere has been shown to drop the cost of treatment significantly by utilising targeted strategies [30]. The limited grazing availability on this farm does not allow much flexibility for pasture rotation. However, continuing with the daily faecal collection, reduced mixing of species and maintaining a stable stocking density could minimise pasture contamination. Monitoring for the presence of clinical signs, predominance of *H. contortus* infection and targeted selective treatment (TST) using the FAMACHA against haemonchosis are likely sustainable preventative animal‐level control strategies in this flock. After noting the initial predominance of *H. contortus* in this outbreak, the FAMACHA method could be used to monitor for ewes suffering from subclinical haemonchosis and targeted use of anthelmintics if further outbreaks occur [1]. FAMACHA card access and online training is now available in the United Kingdom [31] and has supported this farmer, highlighting the need for appropriate diagnosis of predominance of *H. contortus* over other classical PGE from mixed GIN infections, which require different measures for TST and ruling out other differential diagnoses (e.g. fasciolosis) in partnership with their vet. Other complementary control measures, such as genetic selection for host resistance and/or resilience [32], would be unlikely due to the relatively small size of the UK Spotted Dutch sheep breeding population. A commercial *H. contortus* vaccine is available in some countries but is not currently licenced in the United Kingdom. The vaccine was not considered appropriate in this case, as further outbreaks were adequately prevented by diagnostic monitoring and informed pasture management. Hence, regular FWECs combined with species identification at key risk periods have been utilised to inform TST strategies throughout future grazing seasons in this flock.

In conclusion, this case report highlights the benefits of utilising genomic technologies in clinical investigations to monitor for fluctuation in GIN species composition in sheep flocks. We also demonstrate the importance of interpreting species identification results in the context of the flock management history, clinical presentation, standard diagnostic and faecal parasitology results to understand local GIN epidemiology and to inform management strategies.

## Funding

No funding was received for this manuscript.

## Ethics Statement

The study was conducted as part of routine clinical work.

## Conflicts of Interest

The authors declare no conflicts of interest.

## Supporting information


**Supporting Information** Additional supporting information can be found online in the Supporting Information section. Supporting Information. Supporting Material S1: Statistics of deep‐amplicon genomic sequencing of the ITS‐2 region (nemabiome) results used to generate Figure 2. Supporting Material S2: Neighbour‐joining tree of rDNA ITS2 sequences. Constructed using 200 nucleotide sequences of different nematode species downloaded from the NCBI database, along with five *H. contortus* marked with black squares, four *O. venulosum* marked with black circles, five *T. circumcincta* marked with black triangles, two *T. axei* marked with clear squares, two *T. vitrinus* marked with black diamonds and one *T. colubriformis* marked with a clear circle identified in this study. The ASVs clustered closely with their respective reference taxa, confirming accurate taxonomic assignment.

## Data Availability

The data that support the findings of this study are available on request from the corresponding author. The data are not publicly available due to privacy or ethical restrictions.
