# Supplementary material for: Investigating the Epidemiology of Haemonchus contortus, Using Traditional Faecal Diagnostics Supported by Deep‐Amplicon Sequencing, in an Outbreak of Haemonchosis in a Scottish Pedigree Sheep Flock
Source: Case Rep Vet Med. 2026 Aug 6;2026:5558878. doi: 10.1155/crve/5558878 (PMC13444728; doi:10.1155/crve/5558878)
Supplement: Supplementary file 1 [file CRVE-2026-5558878-s001.zip › 5558878.f1/2026.06_Acute-Haemonchosis-sheep-alpaca_SUPP-MAT01_R01.docx]

**Supplementary material**

| **Sample ID** | **Barcode Sequence** | **# Reads** | **Yield (Mbases)** | **Mean Quality Score** | **% Bases >= 30** |
| --- | --- | --- | --- | --- | --- |
| 1 | GCTAGGGT+AAGGACTA | 2,820 | 2 | 32.91 | 80.09 |
| 10 | TAGGCATG+CTAAGCCT | 1,564 | 1 | 34.52 | 86.16 |
| 11 | GTGTGTAG+CTAAGCCT | 12 | 0 | 31.51 | 74.7 |
| 12 | CAGAGAGG+CTAAGCCT | 1,447 | 1 | 34.19 | 85.12 |
| 13 | GCTAGGGT+CTAAGCCT | 142 | 0 | 32 | 76.43 |
| 14 | CGAGGCTG+CTAAGCCT | 19 | 0 | 24.06 | 45.9 |
| 15 | AAGAGGCA+CTAAGCCT | 17 | 0 | 26.64 | 56.19 |
| 16 | GTAGAGGA+CTAAGCCT | 245 | 0 | 29.05 | 65.33 |
| 17 | TAAGGCGA+CGTCTAAT | 2,673 | 2 | 35.07 | 88.64 |
| 18 | CGTACTAG+CGTCTAAT | 2,885 | 2 | 35.16 | 89.04 |
| 19 | AGGCAGAA+CGTCTAAT | 3,864 | 2 | 30.31 | 70.22 |
| 2 | CGAGGCTG+AAGGACTA | 210 | 0 | 28.87 | 64.56 |
| 20 | TCCTGAGC+CGTCTAAT | 2,164 | 1 | 35.09 | 88.87 |
| 21 | GGACTCCT+CGTCTAAT | 1,930 | 1 | 35.09 | 88.81 |
| 22 | TAGGCATG+CGTCTAAT | 2,381 | 1 | 34.87 | 87.72 |
| 23 | GTGTGTAG+CGTCTAAT | 1,172 | 1 | 31.45 | 73.48 |
| 24 | CAGAGAGG+CGTCTAAT | 2,593 | 2 | 34.96 | 88.15 |
| 3 | AAGAGGCA+AAGGACTA | 625 | 0 | 33.04 | 80.53 |
| 4 | GTAGAGGA+AAGGACTA | 9 | 0 | 29.71 | 66.76 |
| 5 | TAAGGCGA+CTAAGCCT | 2 | 0 | 22.81 | 40.7 |
| 6 | CGTACTAG+CTAAGCCT | 5 | 0 | 28.65 | 63.82 |
| 7 | AGGCAGAA+CTAAGCCT | 2,617 | 2 | 34.79 | 87.33 |
| 8 | TCCTGAGC+CTAAGCCT | 1,659 | 1 | 31.54 | 74.83 |
| 9 | GGACTCCT+CTAAGCCT | 4 | 0 | 32.86 | 78.95 |

**Supplementary material 1.** Statistics of deep-amplicon genomic sequencing of the ITS-2 region (nemabiome) results used to generate figure 2.

**Supplementary material 2. Neighbour-joining tree of rDNA ITS2 sequences.** Constructed using 200 nucleotide sequences of different nematode species downloaded from the NCBI database, along with five *H. contortus* marked with black squares, four *O. venulosum* marked with black circles, five *T. circumcincta* marked with black triangles, two *T. axei* marked with clear squares, two *T. vitrinus* marked with black diamonds, and one *T. colubriformis* marked with a clear circle identified in this study. The ASVs clustered closely with their respective reference taxa, confirming accurate taxonomic assignment.
